# Obituary: Yukio Mano (1943–2004)

**DOI:** 10.1186/1743-0003-2-1

**Published:** 2005-02-24

**Authors:** Katsunori Ikoma

**Affiliations:** 1 Department of Rehabilitation and Physical Medicine, Hokkaido University Graduate School of Medicine, Sapporo, Japan

## Abstract

Yukio Mano, MD, PhD (1943–2004)

Associate Editor, Journal of NeuroEngineering and Rehabilitation

I was terribly shocked to hear of the tragic and sudden passing of Yukio Mano on November 7, 2004, at the age of 61. He had not been well this past year but had been working continuously until just ten days before his death.

Yukio Mano (Figure 1) was born on August 26, 1943 in Aichi Prefecture, Japan. He studied medicine at Nagoya University School of Medicine, and graduated in 1968. After he completed his basic medical training in Japan, he began his medical career in the USA in 1972. He first worked as a resident at the Institute of Rehabilitation Medicine at New York University for two years, then in 1974 he moved to the Department of Neurology at Baylor College of Medicine working as an assistant instructor and resident for one year. In 1975, he became a research fellow at the University of Maryland School of Medicine, in the Neuromuscular Research Unit. Yukio Mano studied the most advanced techniques in the fields of rehabilitation medicine and neurology during his four-year stay in the USA. Upon returning to Japan in 1976, he resumed his research in rehabilitation medicine at Nagoya University and the National Center of Neurology and Psychiatry, Japan. In 1981, he became an associate professor in the Department of Neurology at Nara Medical University. He was responsible for running the rehabilitation department there as a specialist in rehabilitation medicine. Finally, he was granted a full professorship in Rehabilitation and Physical Medicine at Hokkaido University (Graduate) School of Medicine in 1995. Yukio Mano was committed to helping researchers studying rehabilitation medicine, as well as young medical doctors and therapists in the rehabilitation field. He extensively expanded the Rehabilitation Department of Hokkaido University, and my colleagues and I had expected his leadership to continue into the future.

His research interest was rehabilitation medicine, especially brain plasticity. He was the first Japanese developer of an apparatus that could deliver transcranial magnetic stimulation. Using this apparatus, he analyzed changes in the central nervous system resulting from various diseases, including brain plasticity after anastomosis of the musculocutaneous and intercostal nerves following cervical root avulsion, and cortical reorganization in training. The knowledge resulting from his research proved beneficial in the rehabilitation of disabled patients. He also emphasized a multidisciplinary approach to rehabilitation medicine and adopted new techniques from engineering. He received the Best Paper Award from ANNIE (Artificial Neural Networks in Engineering) in 2000 for his work entitled, "Adaptive FES Switching System for Hemiplegics".

Yukio Mano served as a council member of the International Society of Electrophysiology and Kinesiology (ISEK) and, in 2000, he organized the XIII Congress of the ISEK in Sapporo, Japan. He was also a member of the editorial board of the Journal of Electromyography and Kinesiology. He served as a council member of many Japanese societies and organized nationwide congresses in Japan even in the year he died. He welcomed the launch of the new Journal of NeuroEngineering and Rehabilitation (JNER) and was honored to be asked to join the Editorial Board as an Associate Editor. He was indeed fully active to his last day.

We cannot help praising him for all that he has accomplished in the fields of rehabilitation medicine, neurophysiology and kinesiology. We must also not forget that this remarkable scientist was also a caring family man. He always showed his love for his family as well as for his colleagues and friends. The loss of such an outstanding personality has been met with great sorrow by his family and the international scientific community. We will always remember him with great affection.

**Figure 1:**
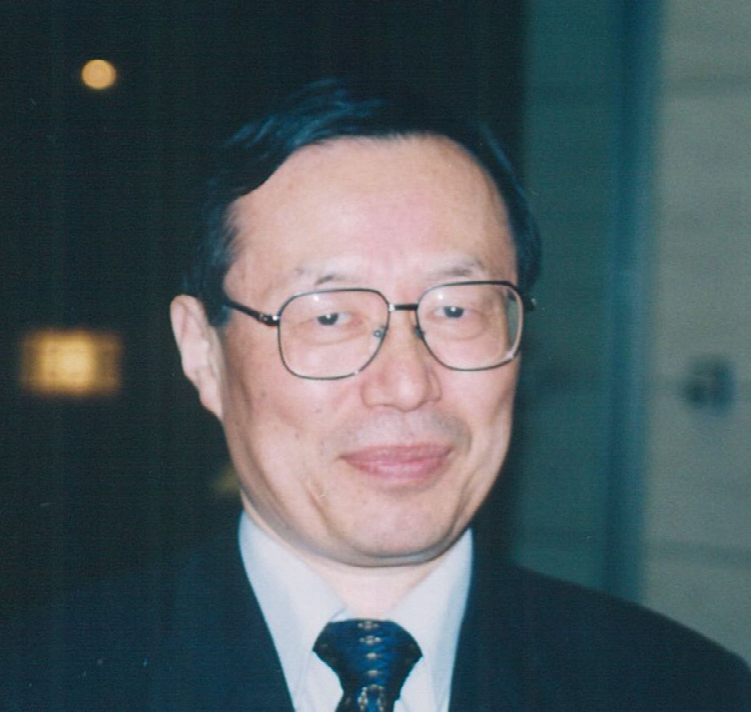
Yukio Mano, MD, PhD 1943–2004

